# Recent advances in the genome-wide study of DNA replication origins in yeast

**DOI:** 10.3389/fmicb.2015.00117

**Published:** 2015-02-19

**Authors:** Chong Peng, Hao Luo, Xi Zhang, Feng Gao

**Affiliations:** ^1^Department of Physics, Tianjin University, Tianjin, China; ^2^Key Laboratory of Systems Bioengineering (Ministry of Education), Tianjin University, Tianjin, China; ^3^SynBio Research Platform, Collaborative Innovation Center of Chemical Science and Engineering, Tianjin, China

**Keywords:** DNA replication, replication origin, *Saccharomyces cerevisiae*, *Schizosaccharomyces pombe*, *Kluyveromyces lactis*, *Pichia pastoris*

## Abstract

DNA replication, one of the central events in the cell cycle, is the basis of biological inheritance. In order to be duplicated, a DNA double helix must be opened at defined sites, which are called DNA replication origins (ORIs). Unlike in bacteria, where replication initiates from a single replication origin, multiple origins are utilized in the eukaryotic genomes. Among them, the ORIs in budding yeast *Saccharomyces cerevisiae* and the fission yeast *Schizosaccharomyces pombe* have been best characterized. In recent years, advances in DNA microarray and next-generation sequencing technologies have increased the number of yeast species involved in ORIs research dramatically. The ORIs in some non-conventional yeast species such as *Kluyveromyces lactis* and *Pichia pastoris* have also been genome-widely identified. Relevant databases of replication origins in yeast were constructed, then the comparative genomic analysis can be carried out. Here, we review several experimental approaches that have been used to map replication origins in yeast and some of the available web resources related to yeast ORIs. We also discuss the sequence characteristics and chromosome structures of ORIs in the four yeast species, which can be utilized to improve yeast replication origins prediction.

## INTRODUCTION

DNA replication is one of the crucial steps for cell cycle. During cell division, accurate and complete duplication of the genome is required to ensure the faithful inheritance of genetic information from one cell generation to the next. To be duplicated, a DNA double helix must be opened at defined sites, termed DNA replication origins (MacAlpine and Bell, 2005; Mechali, 2010; Schepers and Papior, 2010). In general terms, the number of origins (ORIs) in a genome is bound up with the size of the chromosome. Bacterial genomes frequently have a single replication origin, because they usually consist of a small circular chromosome (Gao and Zhang, 2007; Gao et al., 2013; Leonard and Mechali, 2013). In contrast, eukaryotic DNA replication initiates at multiple origins due to their enormous genomic information and the complexity of their chromosome structures (Mechali, 2010). Budding yeast *Saccharomyces cerevisiae* and the fission yeast *Schizosaccharomyces pombe* have the best characterized ORIs in eukaryotes. In *S. cerevisiae*, origin selection is mediated by the formation of a multi-protein complex termed the pre-replicative complex (pre-RC), whose activation leads to DNA unwinding and the assembly of replisomes to carry out DNA synthesis (Bell and Dutta, 2002). Proteins required for pre-RC formation include the origin recognition complex (ORC), the pre-RC assembly factors Cdc6 and Cdt1 and the putative replicative DNA helicase, the MCM2-7 complex (Bell, 2002; Bowers et al., 2004).

Recent advances in DNA microarray technology and next-generation sequencing technologies have brought a dramatic increase in the number of ORIs identified in eukaryotic genomes, such as human (Cadoret et al., 2008; Karnani et al., 2010), mouse (Sequeira-Mendes et al., 2009; Cayrou et al., 2011), *Arabidopsis thaliana* (Costas et al., 2011), and *Drosophila melanogaster* (Cayrou et al., 2011; Gao et al., 2012). The ORIs in some non-conventional yeast species such as *Kluyveromyces lactis* (Liachko et al., 2010) and *Pichia pastoris* (Liachko et al., 2014) have also been genome-widely identified. Because of the increasing data of eukaryotic ORIs, some secondary databases with comprehensive and intuitive ORIs’ information have been constructed. In this review, we summarize several experimental approaches that have been used to identify replication origins in yeast and list some available web resources relevant to yeast ORIs. In addition, we also discuss the characteristics of ORIs in the four yeast species based on the sequence data in the Database of Eukaryotic ORIs (DeOri), including the significant motifs found by the MEME-ChIP web service, the chromosome structures of ORIs, and the origin replication timing and efficiency features.

## EXPERIMENTAL METHODS TO IDENTIFY YEAST REPLICATION ORIGINS

Primal efforts to identify origins across an entire chromosome were two-dimensional gel agarose electrophoresis, which utilized the fact that non-linear DNA molecule does not migrate in gels at the same rate as a linear molecule of equal mass (Bell and Byers, 1983; Brewer and Fangman, 1987). Partially unwound DNA are likely to form only in the vicinity of replication origins, and such structures can be mapped by virtue of being branched. For the relatively low throughput of two-dimensional gel agarose electrophoresis, just a small set of activity origins in the smallest chromosomes in *S. cerevisiae* were located by this method (Reynolds et al., 1989; Newlon et al., 1993; Friedman et al., 1997; Besnard et al., 2014).

To comprehensively identify the location of origins and characterize the ORIs, microarray-based approaches were developed. The combination of fluorescently labeled DNA and microarray representing all the yeast open reading frames (ORFs) can reveal the replicating details of the DNA sequence. Even though they are time consuming and the resolution may not be ideal, these studies make it possible to locate ORIs genome-widely.

There are three widely used microarray-based techniques. (a) By generating a replication timing profile and taking advantage of the fact that ORIs replicate earlier than its neighboring sequences. Methods to differentiate replicated from non-replicated DNA in the progression of DNA replication are diversiform. Both density transfer approach by isotopically labeling of DNA (heavy : light study) and copy number approach by monitoring the change of copy number (Raghuraman et al., 2001; Yabuki et al., 2002; Heichinger et al., 2006) were involved. (b) By identifying pre-replicative complexes at origins of replication using chromatin immunoprecipitation (ChIP). The genome-wide identification of ORC- and MCM-bound sites can reveal the locations of DNA replication origins (Wyrick et al., 2001; Nieduszynski et al., 2006; Xu et al., 2006; Hayashi et al., 2007). (c) By measuring the accumulation of single-stranded DNA (ssDNA) in the presence of a replication-impeding drug, hydroxyurea (HU). This technique makes use of the observation that ssDNA formation is restricted to origins of replication in the checkpoint-deficient mutant *rad*53 (Feng et al., 2006; Masai et al., 2010).

In recent years, the next-generation sequencing technology has also been combined into replication origins identifying methods. Sequencing of replication intermediates or direct sequencing of short, newly replicated DNA strands can help locate replication origins. Compared with microarray-based approaches, deep-sequencing-based approach is characterized by high efficiency, low cost and high resolution. Some methods can even define replication origin sequences throughout the genome with single-nucleotide resolution. On the other hand, next-generation sequencing technologies exhibit coverage biases, which should be avoided to ensure the accuracy of whole-genome origin maps (Besnard et al., 2014).

ChIP-seq, ChIP followed by direct high-throughput sequencing, is the most representative application (Kharchenko et al., 2008). Xu et al. (2012) identified ORIs in three distantly related fission yeasts, *Schizosaccharomyces pombe*, *Schizosaccharomyces octosporus*, and *Schizosaccharomyces japonicas* at high resolution with a generally applicable deep-sequencing-based approach. They counted the frequency of each region of the genome in S-phase arrested cells by deep sequencing, then produced replication timing profiles by mapping all the sites with increased DNA copy number (Xu et al., 2012). Autonomously replicating sequences ARS-seq followed with miniARS-seq is another sequencing-based method. The most recently updated ORIs in *S. cerevisiae* and the firstly reported ORIs in *P. pastoris* are identified with this method (Liachko et al., 2013, 2014). We take *P. pastoris* for instance here to represent the operation steps of this technique. Liachko et al. (2014) firstly constructed a ∼15 × library of genomic DNA in a non-replicating *URA*3 shuttle vector, then screened for ARS activity. ARS inserts were amplified by vector-specific Illumina primers and sequenced by paired-end deep sequencing. Short subfragments of ARSs isolated from the initial ARS-seq screen were then constructed as an input library for a follow-up ARS screen. The subsequent usage of miniARS-seq generated a high-resolution map of ARS sites in the *P. pastoris* genome (Liachko et al., 2014).

**FIGURE 1:**
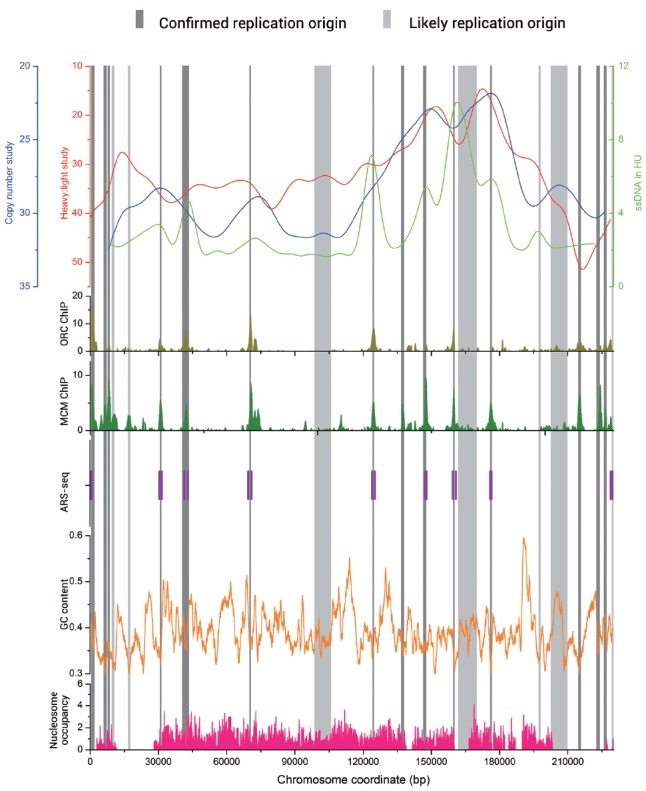
**Graph view of genome-wide data relevant to the replication origins in *Saccharomyces cerevisiae* chromosome 1.** The genome-wide data including heavy: light study (red line), copy number study (blue line), ssDNA in HU study (light green line) are visualized at the top of Figure. The bottom five plots show the genome-wide data of ORC-ChIP (olive bars), MCM-ChIP (green bars), ARS-seq (vertical purple bars), GC content (orange line), and nucleosome occupancy (pink bars), respectively. The replication origin sites are indicated by vertical bars (dark gray for confirmed and light gray for likely).

In Figure 1, we present DNA replication data from different experimental approaches of chromosome 1 in *S. cerevisiae*. The data of microarray-based techniques including heavy : light study, copy number study, ORC-ChIP, and MCM-ChIP, as well as ssDNA in HU study were downloaded from the DNA replication origin database OriDB (Nieduszynski et al., 2007). We also mark the ORIs identified by ARS-seq method on the figure (Liachko et al., 2013). Obvious overlaps exist among the different groups of data.

## DATABASES RELEVANT TO THE STUDY OF YEAST REPLICATION ORIGINS

Due to the increasing data of eukaryotic ORIs, developing repositories of these information became feasible and necessary. We list some of the available web resources relevant to DNA replication in yeast, and discuss their contents in this section.

OriDB^1^ is the most widely used database of DNA replication origins, which is limited to budding yeast (*S. cerevisiae*) and fission yeast (*S. pombe*) by present. The data of *S. cerevisiae* replication origins in OriDB was collated from four microarray-based studies, each of which separately mapped the approximate location of ORIs throughout the yeast genome, and the fifth study that used analysis of phylogenetic conservation and provided another list of origin sites. After amalgamating the data of each study, OriDB produced an integrated list of origin sites. Each proposed origin site is assigned a status (confirmed, likely, or dubious) that indicates the assurance of the site genuinely corresponding to an origin. In 2012, origin sites from *S. pombe* were collected. OriDB provides lots of assistance to researchers working in the DNA replication field because it brings together comprehensive information which was difficult to access and compare (Nieduszynski et al., 2007; Siow et al., 2012).

DeOri^2^ was constructed in the year of 2012 and has been updated constantly. When the original version was constructed, DeOri contained replication origins from six eukaryotic organisms. Now the entries have been increased to 173,988 ORIs from eight eukaryotic organisms, including human, mouse, *A. thaliana*, *D. melanogaster*, *K. lactis*, *S. pombe*, *P. pastoris*, and *S. cerevisiae*. We have filtered the replication origin data in the four yeasts for the following sequence analyzing. This database aims to contribute in the comparative genomic analysis of replication origins, and provides some insights into the nature of replication origins on a genome scale (Gao et al., 2012).

DNAReplication^3^ is a database aimed to provide information and resources for the eukaryotic DNA replication community. Organism-sorted data on replication proteins are presented in this database, and are summarized in the categories of nomenclature, biochemical properties, motifs, interactions, modifications, structure, cell localization and expression, and general comments. Users are also provided with links to recent replication papers, other useful replication websites, and homepages of replication labs. All these functions make this database a valuable tool for the study of eukaryotic DNA replication (Cotterill and Kearsey, 2009).

ReplicationDomain^4^ is a comparative web-based database for storing, sharing and visualizing DNA replication timing data. Other genome-wide chromatin features as well as comparative information of transcriptional expression are also provided in this database. Replication Domain is also a valuable resource for the scientific community because users not only can download the publicly available microarray data, but also are allowed to upload their own data sets and share them with colleagues prior to providing public access (Weddington et al., 2008).

SGD (*Saccharomyces* Genome Database, available at http://www.yeastgenome.org/) is a genomic resource of the budding yeast *S. cerevisiae*. The highest-quality comprehensive information, including the complete *S. cerevisiae* reference genome DNA sequence, its genes and their products, the phenotypes of its mutants, and the literatures supporting these data, are provided in the SGD project (Cherry et al., 2012). ARSs mentioned in peer-reviewed literatures are also integrated in this database. For each ARS, the details about its sequence, location, relative literatures, and history can be obtained. Users can also use the analysis tools such as BLAST provided in SGD to explore these data.

## SEQUENCE CHARACTERISTICS OF YEAST REPLICATION ORIGINS

In budding yeast *S. cerevisiae*, replication origins are defined as ARS because they can support the maintenance of a plasmid in growing yeast cells (Stinchcomb et al., 1979). Every replication origin contains a conserved 11-bp motif (sometimes assigned as 17 bp in length) called the ARS consensus sequence (ACS) that is essential for the binding of the initiator protein ORC (Rao and Stillman, 1995; Rowley et al., 1995; Theis and Newlon, 1997). A match to the ACS is essential but not sufficient for origin function. Even though, some bioinformatic algorithms for predicting the location of yeast replication origins have been developed based on ACS. For example, to predict the location of ORIs in the *S. cerevisiae* genome, Breier et al. (2004) developed an algorithm called Oriscan. This method utilized 268 bp of sequence, including the T-rich ACS and a 3′ A-rich region to identify ORI candidates. It then ranked potential origins by their likelihood of activity. A large proportion of origins in the genome were recognized by Oriscan with near-perfect specificity (Breier et al., 2004). Another computational study made use of the discovery that most replication origin sequences are phylogenetically conserved among closely related *Saccharomyces* species. It combined motif searches, phylogenetic conservation, and microarray data together to identify replication origin sequences throughout the *S. cerevisiae* genome (Nieduszynski et al., 2006). Analogously, the ORIs in *K. lactis* also contain a 50-bp ACS. The difference is that ACS in *K. lactis* ARSs is both necessary and largely sufficient for ARS activity (Liachko et al., 2010).

Abundant research was also conducted on the replication origins in fission yeast *S. pombe*, where replication sequences also function as autonomous replicators. However, ORIs in *S. pombe* do not have recognizable consensus elements but have a 500–1000 bp extended AT-rich structure (Dubey et al., 1994; Clyne and Kelly, 1995). Segurado et al. (2003) identified 384 potential origins by this feature. It was previously believed that replication origins in plant and metazoan are G/C-rich while in yeasts are A/T-rich. However, an industrially important methylotrophic budding yeast, *P. pastoris*, owed different characteristics in its ORIs compared with other studied yeasts. In this kind of yeast, two different types of ORIs exist simultaneously. In addition to an A/T-rich type more reminiscent of typical budding and fission yeast origins, there is also a G/C-rich type of replication origins associated with transcription start sites (Liachko et al., 2014). We calculate the GC content along *S. cerevisiae* chromosome 1 with sliding window algorithm (window size: 1000, shift: 20) and present it in Figure 1 by the orange line. This line indicates that GC contents of the ORIs sequences are significantly lower than those of the entire genome sequences. In fact, this status exists in all the four kinds of yeasts, even in *P. pastoris*, the one includes G/C-rich type of ORIs.

**FIGURE 2:**
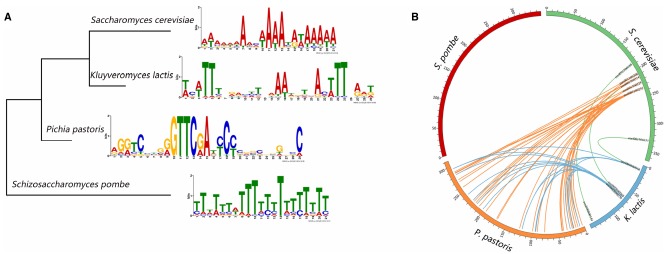
**Sequence characteristics of yeast replication origins. (A)** The significant motifs found in the replication origin sequences and the phylogenetic tree of the four yeasts. **(B)** The circos plot of replication origins that share similar sequences. Each number around the circle is the ORI’s serial number in DeOri.

To gain a comprehensive view of the conserved motifs in the origin sequences, we use the MEME-ChIP web service to discovery enriched motifs in the ORI sequences in the four kinds of yeasts. MEME-ChIP web service is designed especially for discovering motifs in the large sets of short DNA sequences (Bailey et al., 2009; Machanick and Bailey, 2011). The motifs we found are displayed in Figure 2A. ORIs in *S. cerevisiae*, *K. lactis*, and *S. pombe* contain AT-rich motifs, whereas GC-rich motifs are found in *P. pastoris* ORIs. We also construct the phylogenetic tree (Figure 2A) of the four organisms based on the cytochrome c downloaded from NCBI. The tree was constructed using the MEGA6 program (Statistical Method: Maximum Likelihood, Test of Phylogeny: Bootstrap method, No. of Bootstrap Replications: 1000; Tamura et al., 2013). Conserved motifs found in the four yeasts ORIs show no significant correlation with their phylogenetic relationships.

In addition, regions of local similarity in sequences between each pair of organisms are searched by the BLAST program (Altschul et al., 1997). Figure 2B is created by circos (Krzywinski et al., 2009), and shows the ORIs that share similar sequences. Each number around the circle is the ORI’s serial number in DeOri. When two ORIs share similar local regions, a line will be drawn between them. For example, eori001300188, eori001300214, and eori001300331 have local regions similar with eori000800141, eori000800068, and eori000800010, respectively, hence the three pairs of ORIs are connected. No significant similarity is found between sequences in *S. pombe* ORIs and any other three groups of sequences. This may be caused by the large phylogenetic distance of *S. pombe*.

A new study suggests that in budding yeast, specific origin sequences are not strictly required for DNA replication *in vitro*, although they are essential for plasmid replication *in vivo*. The observation supports the notion that DNA replication specification in budding yeast is not completely dependent on DNA sequences, and epigenetic mechanisms are also important for determining replication origin sites (Gros et al., 2014).

## DISTRIBUTION AND ORGANIZATION OF YEAST REPLICATION ORIGINS

Despite the lack of uniform feature of replication origin sequences, ORIs do not randomly locate on chromosome. Indeed, in all the four kinds of yeasts, origins have a significant preference for intergenic regions (Hayashi et al., 2007; Liachko et al., 2010, 2014; Renard-Guillet et al., 2014). We find that the correlation coefficient values (R values) between the chromosome length and replication origins number are 0.956, 0.999, 0.966, and 0.854 for *S. cerevisiae*, *S. pombe*, *K. lactis*, and *P. pastoris*, respectively, which indicates that longer chromosomes tend to have more ORIs. In addition, ORIs always appear in the nucleosome-free regions (Li et al., 2014; Sherstyuk et al., 2014). We collect the nucleosome occupancy data in *S. cerevisiae* chromosome 1 (Kaplan et al., 2009) and map it in Figure 1 by pink bars. The nucleosome occupancy scores in ORIs are significantly lower, which agrees well with the above conclusions. An asymmetric pattern of positioned nucleosomes has been verified at origins in both *S. cerevisiae* and *K. lactis* (Eaton et al., 2010; Tsai et al., 2014). These nucleosome occupancy information has been successfully used to train a machine learning algorithm to predict the position of active arm origins in the *Candida albicans* genome (Tsai et al., 2014).

Two other important features of ORIs are origin replication timing and efficiency. Origins are fired at various time throughout the S phase. *S. cerevisiae* ORIs can be separated into early and late origins. They present different nucleosomal architectures, which are already established in G1 phase. A higher occupancy of nucleosomes and broader nucleosome-depleted region (NDR) features appear in early origins, while late origins display a lower occupancy and tighter NDR (Soriano et al., 2014). In *S. pombe*, early and late origins tend to distribute separately in large chromosome regions (Hayashi et al., 2007). The dynamics of replication in *P. pastoris* shows an unexpected difference in replication timing between GC-ARSs and AT-ARSs. GC-rich ORIs replicate remarkably earlier and/or more efficiently than AT-rich ORIs (Liachko et al., 2014). In regard to origin replication efficiency, not all origins are used at each cell cycle. The overall efficiency of origin firing is less than 50% in *S. cerevisiae* and *S. pombe* (Friedman et al., 1997; Heichinger et al., 2006). It appears to be that the replication stress presented by different growth conditions affects the number of sites being activated (Tuduri et al., 2010). The flexibility of replication origins may be an obstacle in the thorough genome-wide understanding of ORIs in yeast.

### CONFLICT OF INTEREST STATEMENT

The authors declare that the research was conducted in the absence of any commercial or financial relationships that could be construed as a potential conflict of interest.

## Footnotes

1 http://cerevisiae.oridb.org/

2 http://tubic.tju.edu.cn/deori/

3 http://www.dnareplication.net/

4 http://www.replicationdomain.org
